# Prevalence of Highly Sensitive Personality and Its Relationship With Depression, and Anxiety in the Saudi General Population

**DOI:** 10.7759/cureus.49834

**Published:** 2023-12-02

**Authors:** Mohammed Dosari, Saud K AlDayel, Khalid M Alduraibi, Abdulaziz A AlTurki, Fahad Aljehaiman, Sultan Alamri, Hamad S Alshammari, mosad Alsuwailem

**Affiliations:** 1 Family Medicine, King Abdulaziz Medical City Riyadh, Riyadh, SAU; 2 Medical School, King Saud Bin Abdulaziz University for Health Sciences College of Medicine, Riyadh, SAU

**Keywords:** sensory processing sensitivity, general population, depression, anxiety, highly sensitive personality

## Abstract

Background

Highly sensitive personality (HSP) occurs in those who experience stronger processing of emotions and responses to both internal and external stimuli; this, in turn, could cause persons with highly sensitive personalities to suffer from affective disorders such as depression and anxiety at higher rates. This study aimed to measure the prevalence of highly sensitive personality and its relationship with depression and anxiety among the Saudi general population.

Subject and methods

This cross-sectional study was conducted among the general population in Saudi Arabia. A self-administered questionnaire was sent to the target population using an online survey. The questionnaire includes sociodemographic data (i.e., age, gender, marital status, etc.), the Highly Sensitive Person Scale to measure the degree of sensitivity, and the Hospital Anxiety and Depression Scale (HADS) to measure anxiety and depression.

Results

Of the 438 participants, 72.6% were females, and 48.9% were aged between 18 to 25 years. The prevalence of HSP in this study was 29%. Abnormal levels of anxiety and depression were found in 29.5% and 19.9%, respectively. Significant relationships were observed between HSP in terms of anxiety (p<0.001) and depression (p=0.001). It is interesting to note that a previous diagnosis of mental disorder was identified as a significant risk factor for HSP, anxiety, and depression.

Conclusion

There was a high prevalence of HSP in our population, which was significantly associated with anxiety and depression. Further, female participants were more likely to exhibit HSP and anxiety but not depression. These findings highlight the importance of prevention programs intended for highly sensitive persons with associated mental conditions.

## Introduction

Highly sensitive personality (HSP) or sensory processing sensitivity (SPS) is a hereditary personality trait that is associated with a genetic component leading to deep processing and response to external stimuli [1,2]. The external stimuli can involve varied aspects such as noises, caffeine, pain, and hunger sensations. Also, it can affect other parts of the person's life such as their personal relationship, work colleagues, and school peers [2-4]. People with HSP have a higher awareness of their surroundings and the moods of others which can lead to more empathetic responses due to their deep processing [5,6]. However, this trait of deep thinking and increased empathy can lead to absorbing other people's emotions and this can become overwhelming for them [7]. In addition, people with HSP have higher rates of neuroticism and introversion compared to the general population, which can put them at a higher risk for some affective disorders [8]. According to Aron (2010), it has been conducted that around half of patients who complain of shyness as well as anxiety and depressive symptoms have HSP [9]. 

In recent years many researchers have made a correlation between highly sensitive personality and mood disorders such as depression and anxiety. A study performed on university students in 2018 showed there’s a direct relation between overstimulation in highly sensitive persons and depressive tendencies [10]. It is important to mention the factors that come into play when discussing affective disorders and HSP. For instance, environmental factors play a key role; depression and anxiety have been linked with high sensory processing sensitivity (SPS) which was primarily found in individuals subjected to negative childhood experiences [11]. Further supporting this claim is a study using a shortened version of the Highly Sensitive Person Scale (HSPS) which categorized patients’ responses into three factors: ease of excitation (EOE), low sensory threshold (LST), and aesthetic sensitivity (AE). This study proved that there’s a positive correlation between EOE and LST with the degree of psychological health complaints [12]. How external and internal stimuli are perceived has also been shown to increase the risk of developing anxiety in adults, according to a study conducted in Australia using HSPS [13].

This study aims to determine the prevalence and relationship between highly sensitive personality, depression, and anxiety among the general population of Saudi Arabia and recognize the factors that lead to their development. Moreover, this study will be helpful in increasing the level of awareness and knowledge among the general population about these personality traits and how they influence other factors in persons’ lives. This study will also be beneficial for healthcare practitioners in identifying HSP and associated disorders and implementing the best management options for these patients. 

## Materials and methods

This cross-sectional study was conducted with participants from the general population of Saudi Arabia. The co-investigators collected the data using a self-administered questionnaire. The population included in this study were both female and male, aged 18 years and above, ranging from different socioeconomic backgrounds, education levels, and occupations. However, nationality was not specified in the questionnaire. A non-probability convenience sampling technique was used by including all those who met our criteria. The total number of participants in the study was 438.

Participation in the study was voluntary; collection of data ceased when the sample size of 438 was fulfilled. Collection of the data was through an online questionnaire and consent to use the data was taken before participating in the study. Approval of the study was granted by King Abdullah International Medical Research Center (KAIMRC) and Institutional Review Board approval (IRB) was issued thereafter (study number: NRC23R/362/06; IRB Approval No.: IRB/1564/23; E-CTS Ref No.: RYD-23-419812-96052; approval date: June 27, 2023).

HSP was measured by using the Highly Sensitive Person Questionnaire (HSP Scale) developed by Aron and Aron [14]. This is a 27-item questionnaire measuring the sensitive personality of an individual; those scoring 14 and above were considered highly sensitive. Likewise, the anxiety and depression of the general population were measured with the Hospital Anxiety and Depression Scale (HADS) developed by Zigmond and Snaith [15]. This is a validated questionnaire measuring the level of anxiety and depression of the subject consisting of 14 items (seven items each), a four-point Likert scale with categories ranging from 0 to 3 points. Scores of 7 and 10 points use cutoff points between normal, borderline, and abnormal.

Categorical variables were shown as numbers and percentages. Continuous variables were calculated and given as mean and standard deviation. The relationship between the levels of HSP, anxiety, and depression in terms of socio-demographic data collected was determined using the chi-square test. Based on the significant results, multivariate regression models were performed to determine the independent significant predictors associated with high levels of HSP, as well as abnormal levels of anxiety and depression. Statistical significance was set to p<0.05. All data analyses were carried out using the IBM SPSS v. 26 (IBM Corp., Armonk, NY).

## Results

This cross-sectional survey recruited 438 participants. As described in Table 1, the most common age group was 18 to 25 years (48.9%), with females being dominant (72.6%). Participants who lived in the Central Region constituted 38.8%. Most of the respondents were bachelor's degree holders (67.1%). Respondents who were currently employed constituted 25.3%. Among those working (n=266), 75.6% were government employees, mostly in a non-healthcare institution (64.7%). Of the healthcare employees (n=94), 22.3% were doctors. With respect to monthly income, 57.1% were earning less than 10,000 SAR per month. With respect to marital status, 53.9% had never been married.

**Table 1:** Socio-demographic characteristics of participants (n=438)

Study variables	N (%)
Age group in years	
18-25 years	214 (48.9%)
26-35 years	67 (15.3%)
36-50 years	76 (17.4%)
>50 years	81 (18.5%)
Gender	
Male	120 (27.4%)
Female	318 (72.6%)
Residence region	
Central Region	170 (38.8%)
Eastern Region	119 (27.2%)
Northern Region	45 (10.3%)
Southern Region	12 (02.7%)
Western Region	92 (21.0%)
Educational level	
Intermediate school	2 (0.50%)
Secondary school	103 (23.5%)
Bachelor's degree	294 (67.1%)
Master's degree	31 (07.1%)
PhD degree	8 (01.8%)
Employment status	
Employed	111 (25.3%)
Unemployed	81 (18.5%)
Retired	75 (17.1%)
Student	171 (39.0%)
Job type (n=266)	
Government employee	201 (75.6%)
Non-government employee	65 (24.4%)
Institution (n=266)	
Healthcare	94 (35.3%)
Non-healthcare	172 (64.7%)
If you are a health employee, choose your job (n=94)	
Unspecified	55 (58.5%)
Doctor	21 (22.3%)
Nurse	09 (09.6%)
Specialist	05 (05.3%)
Pharmacist	02 (02.1%)
Dentist	02 (02.1%)
Monthly income (SAR)	
<10,000	250 (57.1%)
10,001 – 20,000	116 (26.5%)
20,001 – 30,000	40 (09.1%)
>30,000	32 (07.3%)
Marital status	
Single	236 (53.9%)
Married	180 (41.1%)
Divorced	19 (04.3%)
Widowed	03 (0.70%)

**Figure 1:**
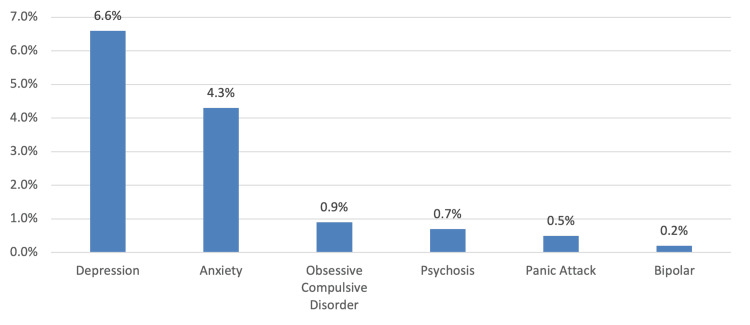
Previous history of mental disorder

In Figure 1, the most commonly diagnosed mental disorders were depression (6.6%) and anxiety (4.3%).

**Figure 2:**
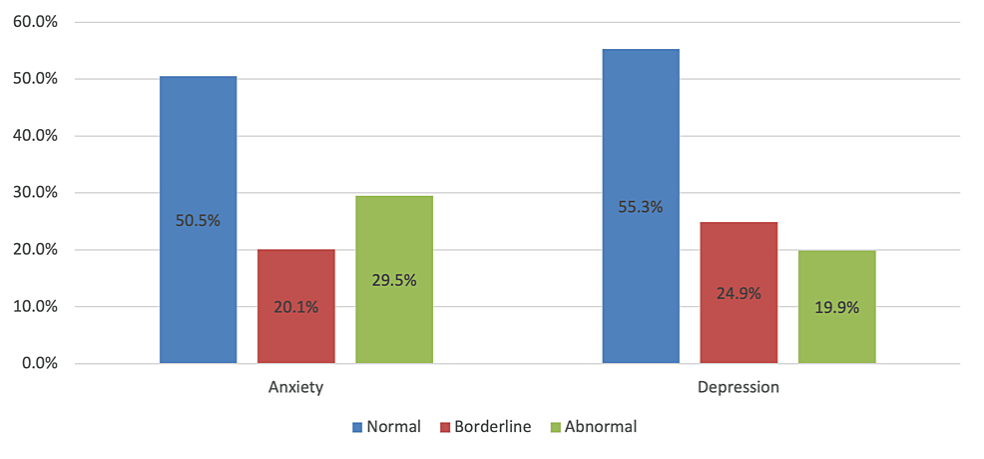
Level of anxiety and depression

The prevalence of highly sensitive personality, depression, and anxiety has been elaborated in Table 2. It can be observed that the total mean score for HSP was 23.7 (SD 3.86), with 29% considered as highly sensitive and the rest were normal (71%). Regarding anxiety, the total mean score was 7.89 (SD 4.65), with 29.5% classified as abnormal levels. Regarding depression, the total mean score was 7.09 (SD 4.00), with 19.9% considered abnormal levels (see also Figure 2).

**Table 2:** Prevalence of highly sensitive personality, depression, and anxiety among the general population (n=438)

Variables	N (%)
HSP total score (mean ± SD)	23.7 ± 3.86
HSP level	
Highly sensitive	127 (29.0%)
Normal	311 (71.0%)
Anxiety score (mean ± SD)	7.89 ± 4.65
Anxiety level	
Normal	221 (50.5%)
Borderline	88 (20.1%)
Abnormal	129 (29.5%)
Depression score (mean ± SD)	7.09 ± 4.00
Depression level	
Normal	242 (55.3%)
Borderline	109 (24.9%)
Abnormal	87 (19.9%)

When measuring the relationship between HSP in terms of anxiety and depression levels (Table 3), it was found that a highly sensitive personality was significantly more common among those with abnormal levels of anxiety (p<0.001) and depression (p=0.001).

**Table 3:** Relationship between highly sensitive personality according to anxiety and depression (n=438) § P-value has been calculated using a chi-square test. ** Significant at p<0.05 level.

Factor	Level of HSP		P-value ^§^
	Highly N (%) ^(n=127)^	Normal N (%) ^(n=311)^	
Anxiety			
Normal	35 (27.6%)	186 (59.8%)	<0.001 **
Borderline	23 (18.1%)	65 (20.9%)	
Abnormal	69 (54.3%)	60 (19.3%)	
Depression			
Normal	59 (46.5%)	183 (58.8%)	0.001 **
Borderline	29 (22.8%)	80 (25.7%)	
Abnormal	39 (30.7%)	48 (15.4%)	

We used the chi-square test in Table 4 to determine the relationship between the HSP level and the participants' socio-demographic characteristics. It was revealed that the prevalence of highly sensitive personality was significantly more common among the younger age group (p=0.003), female gender (p<0.001), lower monthly income (p<0.001), never been married (p=0.001), and those with a previous diagnosis of mental disorder (p<0.001).

**Table 4:** Relationship between the level of HSP and sociodemographic characteristics of participants § P-value has been calculated using chi-square test. ** Significant at p<0.05 level.

Factor	Level of HSP		P-value ^§^
	Highly N (%) ^(n=127)^	Normal N (%) ^(n=311)^	
Age group in years			
≤30 years	86 (67.7%)	163 (52.4%)	0.003 **
>30 years	41 (32.3%)	148 (47.6%)	
Gender			
Male	18 (14.2%)	102 (32.8%)	<0.001 **
Female	109 (85.8%)	209 (67.2%)	
Educational level			
Secondary or below	29 (22.8%)	76 (24.4%)	0.721
Bachelor or higher	98 (77.2%)	235 (75.6%)	
Employment status			
Employed	30 (23.6%)	81 (26.0%)	0.067
Unemployed/Retired	37 (29.1%)	119 (38.3%)	
Student	60 (47.2%)	111 (35.7%)	
Monthly income (SAR)			
<10,000	90 (70.9%)	160 (51.4%)	<0.001 **
≥10,000	37 (29.1%)	151 (48.6%)	
Marital status			
Never been married	84 (66.1%)	152 (48.9%)	0.001 **
Been married	43 (33.9%)	159 (51.1%)	
Previous diagnosis of mental disorder			
Yes	33 (26.0%)	25 (08.0%)	<0.001 **
No	94 (74.0%)	286 (92.0%)	

We also used the chi-square test to determine the relationship between the anxiety level and participants' socio-demographics (Table 5). It was found that the prevalence of abnormal anxiety levels was significantly more common among women (p=0.002), students (p=0.002), unmarried persons (p<0.001) and those under 30 years of age (p<0.001), earning under 10,000 SAR a month (p=0.001), and with a previous diagnosis of mental disorder (p=0.001).

**Table 5:** Relationship between the level of anxiety and sociodemographic characteristics of participants (n=438) § P-value has been calculated using the chi-square test. ** Significant at p<0.05 level.

Factor	Level of anxiety			P-value ^§^
	Normal N (%) ^(n=221)^	Borderline N (%) ^(n=88)^	Abnormal N (%) ^(n=129)^	
Age group in years				
≤30 years	100 (45.2%)	60 (68.2%)	89 (69.0%)	<0.001 **
>30 years	121 (54.8%)	28 (31.8%)	40 (31.0%)	
Gender				
Male	77 (34.8%)	15 (17.0%)	28 (21.7%)	0.002 **
Female	144 (65.2%)	73 (83.0%)	101 (78.3%)	
Educational level				
Secondary or below	48 (21.7%)	21 (23.9%)	36 (27.9%)	0.425
Bachelor or higher	173 (78.3%)	67 (76.1%)	93 (72.1%)	
Employment status				
Employed	62 (28.1%)	18 (20.5%)	31 (24.0%)	0.002 **
Unemployed/Retired	93 (42.1%)	27 (30.7%)	36 (27.9%)	
Student	66 (29.9%)	43 (48.9%)	62 (48.1%)	
Monthly income (SAR)				
<10,000	106 (48.0%)	59 (67.0%)	85 (65.9%)	0.001 **
≥10,000	115 (52.0%)	29 (33.0%)	44 (34.1%)	
Marital status				
Never been married	91 (41.2%)	55 (62.5%)	90 (69.8%)	<0.001 **
Been married	130 (58.8%)	33 (37.5%)	39 (30.2%)	
Previous diagnosis of mental disorder				
Yes	21 (09.5%)	08 (09.1%)	29 (22.5%)	0.001 **
No	200 (90.5%)	80 (90.9%)	100 (77.5%)	

We also used the chi-square test in Table 6 to find the relationship between the level of depression and the sociodemographic characteristics of participants. It was revealed that the prevalence of abnormal depression levels was significantly more common among the younger age group (p<0.001), students (p<0.001), those who had never been married (p<0.001), and previous diagnoses of mental disorder (p<0.001).

**Table 6:** Relationship between the level of depression and sociodemographic characteristics of participants (n=438) § P-value has been calculated using the chi-square test. ** Significant at p<0.05 level.

Factor	Level of depression			P-value ^§^
	Normal N (%) ^(n=242)^	Borderline N (%) ^(n=109)^	Abnormal N (%) ^(n=87)^	
Age group in years				
≤30 years	114 (47.1%)	73 (67.0%)	62 (71.3%)	<0.001 **
>30 years	128 (52.9%)	36 (33.0%)	25 (28.7%)	
Gender				
Male	75 (31.0%)	23 (21.1%)	22 (25.3%)	0.140
Female	167 (69.0%)	86 (78.9%)	65 (74.7%)	
Educational level				
Secondary or below	57 (23.6%)	30 (27.5%)	18 (20.7%)	0.524
Bachelor or higher	185 (76.4%)	79 (72.5%)	69 (79.3%)	
Employment status				
Employed	63 (26.0%)	28 (25.7%)	20 (23.0%)	<0.001 **
Unemployed/Retired	108 (44.6%)	26 (23.9%)	22 (25.3%)	
Student	71 (29.3%)	55 (50.5%)	45 (51.7%)	
Monthly income (SAR)				
<10,000	131 (54.1%)	66 (60.6%)	53 (60.9%)	0.383
≥10,000	111 (45.9%)	43 (39.4%)	34 (39.1%)	
Marital status				
Never been married	99 (40.9%)	74 (67.9%)	63 (72.4%)	<0.001 **
Been married	143 (59.1%)	35 (32.1%)	24 (27.6%)	
Previous diagnosis of mental disorder				
Yes	17 (07.0%)	23 (21.1%)	18 (20.7%)	<0.001 **
No	225 (93.0%)	86 (78.9%)	69 (79.3%)	

We also conducted multivariate regression analyses to identify the independent risk factors for high levels of HSP and abnormal levels of anxiety and depression (Table 7). In the HSP model, it was revealed that female gender and previous diagnosis of mental disorders were identified as the significant risk factors for highly sensitive personality, while higher monthly income was identified as the preventive factor. This further suggests that compared to males, females were 2.69 times more likely to exhibit HSP (AOR=2.686; 95% CI=1.514-4.762; p=0.001). Participants who were diagnosed with mental disorders were 3.85 times more likely to be associated with HSP than those who did not have the diagnosis (AOR=3.855; 95% CI=2.117-7.022; p<0.001). On the contrary, respondents who were high-earners had a decreased risk of being HSP by at least 44% as compared to respondents who were earning less (AOR=0.564; 95% CI=0.346-0.920; p=0.022). In the anxiety model, it was observed that a previous diagnosis of mental disorder was identified as the independent significant risk factor for anxiety while having been married was identified as the significant protective factor. This further indicates that respondents who were previously diagnosed with mental disorders were predicted to increase the risk for anxiety by at least 2.74 times higher (AOR=2.743; 95% CI=1.526-4.933; p=0.001), while respondents who had been married were predicted to decrease the risk of anxiety by at least 62% (AOR=0.383; 95% CI=0.179-0.822; p=0.014). Finally, in the depression model, a previous diagnosis of a mental disorder was also identified as the significant risk factor for depression, whereas having been married was identified as the significant protective factor. This further indicates that respondents who were diagnosed with mental disorders were 1.99 times more likely to be associated with the risk of depression (AOR=1.999; 95% CI=1.063-3.759; p=0.032), while respondents who had been married were predicted to decrease the risk of depression by at least 62% as compared to those who had never been married (AOR=0.379; 95% CI=0.157-0.914; p=0.031).

**Table 7:** Multivariate regression analysis to determine the significant independent predictors of HSP as well as abnormal levels of anxiety and depression (n=438) AOR: adjusted odds ratio; CI: confidence interval; SAR: Saudi Arabian Riyal; Ref: reference ** Significant at p<0.05 level.

Parameters		AOR	95% CI	P-value
HSP Model				
Age group in years	≤30 years	Ref		
	>30 years	0.932	0.438 – 1.983	0.855
Gender	Male	Ref		
	Female	2.686	1.514 – 4.762	0.001 **
Monthly income (SAR)	<10,000	Ref		
	≥10,000	0.564	0.346 – 0.920	0.022 **
Marital status	Never been married	Ref		
	Been married	0.641	0.309 – 1.331	0.233
Previous diagnosis of mental disorder	Yes	3.855	2.117 – 7.022	<0.001 **
	No	Ref		
Anxiety Model				
Age group in years	≤30 years	Ref		
	>30 years	1.012	0.447 – 2.293	0.977
Gender	Male	Ref		
	Female	1.355	0.818 – 2.247	0.238
Employment status	Employed	Ref		
	Unemployed/retired	0.73	0.375 – 1.424	0.356
	Student	0.959	0.505 – 1.820	0.898
Monthly income (SAR)	<10,000	Ref		
	≥10,000	0.801	0.491 – 1.305	0.372
Marital status	Never been married	Ref		
	Been married	0.383	0.179 – 0.822	0.014 **
Previous diagnosis of mental disorder	Yes	2.743	1.526 – 4.933	0.001 **
	No	Ref		
Depression Model				
Age group in years	≤30 years	Ref		
	>30 years	1.031	0.404 – 2.628	0.95
Employment status	Employed	Ref		
	Unemployed/retired	0.939	0.450 – 1.961	0.868
	Student	1.17	0.568 – 2.408	0.67
Marital status	Never been married	Ref		
	Been married	0.379	0.157 – 0.914	0.031 **
Previous diagnosis of mental disorder	Yes	1.999	1.063 – 3.759	0.032 **
	No	Ref		

## Discussion

This study is carried out to examine the prevalence and relationship between HSP, depression, and anxiety among the Saudi general population. The prevalence of HSP in this study was 29%, which was higher than the estimated prevalence of 15% to 20% [3]. Furthermore, the rate of HSP was higher among the younger age group, female gender, lower economic status, single participants, and previous diagnosis of mental disorders. However, in our adjusted regression model, female gender and previous diagnosis of mental disorders were identified as the significant risk factors for HSP, whereas higher monthly income was identified as an HSP preventive factor. This is almost consistent with the study of Pérez-Chacón et al. [16] Based on their reports, women were more associated with higher SPS scores and poorer health-related quality of life than men. Also, they observed that conscientiousness, extraversion, and adaptive coping strategies were determined as protective factors, while maladaptive coping techniques and neuroticism were also identified as risk factors. However, Listou Grimen and Diseth [12] documented that SPS was positively associated with neuroticism and openness but inversely associated with extraversion. Additionally, subjective health complaints (SHC) are greatly influenced by the personality traits of neuroticism more than SPS factors.

Studies suggest an association between HSP and psychological disorders. For instance, Yano and Oishi, [10] reported that low sensory threshold (LST) and ease of excitation (EOE) were associated with depressive symptoms, while aesthetic sensitivity (AES) was inversely associated with depressive tendencies. Similarly, Liss et al. [17] EOE and LST were linked to autism symptoms, anxiety, depression, and alexithymia. AES was linked to the symptoms of autism and anxiety but not to depression. On the other hand, Grinapol et al. [18] found a positive correlation between SPS and post-traumatic stress symptoms (PTSS) at follow-up. However, SPS was not seen to influence PTSS at follow-up when controlled for early PTSS. Likewise, Pluess and Boniwell [19] revealed that SPS was a significant factor in treatment response, wherein the prevention program effectively reduced depression among girls with HSP but was ineffective among girls scoring low on the same measure. In our study, however, HSP was significantly related to anxiety and depression, with more than half of HSP respondents (54.3%) detected to have abnormal anxiety levels (p<0.001), while less than one-third (30.7%) had abnormal depression (p=0.001).

Conversely, a mediation analysis documented by Brindle et al. [7] showed that SPS and depression were mediated by the lack of individual access to emotional regulation strategies, higher emotional awareness, and insufficient acceptance of feeling distressed. In addition, SPS and anxiety and stress symptoms were also mediated partially by the blends of these variables. Another study conducted by Benham [20] disclosed that the inclusion of an interaction term in the model proved to be non-significant, indicating that an additive model best explains the associations between SPS, stress, and health. However, in our study, having been married was associated with improved mental conditions, particularly with anxiety and depression, but not with HSP.

It is important to note that anxiety and depression were also common in our population. The rate of abnormal levels was 29.5% and 19.9% for anxiety and depression, respectively. Influencing factors of anxiety and depression include age group, employment status, marital status, and previous history of mental disorder. However, in our regression estimates, only a previous history of mental disorder was determined as the significant risk factor for both mental conditions. In Spain [21], healthcare workers were seen to have an increased personal realization and depersonalization, while educators demonstrated higher compassion fatigue. In addition, they cited that certain characteristics of SPS had driven the occurrence of burnout and compassion fatigue. Another survey done among Japanese university students [10] reported that the association between depressive symptoms and LST or EOE was mediated by regular physical exercise. The authors suggested that this effect was not conclusive and that longitudinal studies are needed to determine its cause and effect.

Limitations

This study has a few limitations. First, the self-reporting nature of the study may introduce the possibility of response bias. Second, the study was directed towards the Saudi population, generalizing the findings of this study might not be applicable due to cultural and social differences. Third, the study’s cross-sectional design limits its ability to establish casual relationships. On the contrary, this questionnaire was the first in the region to assess the prevalence of HSP and its relationship with depression and anxiety.

## Conclusions

Highly sensitive personality was prevalent among our population. HSP significantly influences psychological conditions, including anxiety and depression. Further, female participants tended to have a highly sensitive personality regardless of mental conditions, while participants who had been married demonstrated improved mental conditions but not HSP. However, a previous diagnosis of mental disorder was the most significant risk factor in all three models used throughout. This study provides evidence of an association between HSP and abnormal mental conditions. However, given the scarcity of this study discipline in our region, more prospective studies are required to establish their associations.

## References

[1] Assary E, Zavos HM, Krapohl E, Keers R, Pluess M (2021). Genetic architecture of Environmental Sensitivity reflects multiple heritable components: a twin study with adolescents. Mol Psychiatry.

[2] Pluess M (2015). Individual differences in environmental sensitivity. Child Dev Perspect.

[3] Aron EN, Aron A, Jagiellowicz J (2012). Sensory processing sensitivity: a review in the light of the evolution of biological responsivity. Pers Soc Psychol Rev.

[4] Jagiellowicz J, Xu X, Aron A, Aron E, Cao G, Feng T, Weng X (2011). The trait of sensory processing sensitivity and neural responses to changes in visual scenes. Soc Cogn Affect Neurosci.

[5] Acevedo BP, Aron EN, Aron A, Sangster MD, Collins N, Brown LL (2014). The highly sensitive brain: an fMRI study of sensory processing sensitivity and response to others' emotions. Brain Behav.

[6] Acevedo B, Aron E, Pospos S, Jessen D (2018). The functional highly sensitive brain: a review of the brain circuits underlying sensory processing sensitivity and seemingly related disorders. Philos Trans R Soc Lond B Biol Sci.

[7] Brindle K, Moulding R, Bakker K, Nedeljkovic M (2015). Is the relationship between sensory‐processing sensitivity and negative affect mediated by emotional regulation?. Aust J Psychol.

[8] Ueno Y, Takahashi A, Oshio A (2019). Relationship between sensory-processing sensitivity and age in a large cross-sectional Japanese sample. Heliyon.

[9] McManus H (2012). Psychotherapy and the highly sensitive person. Improving outcomes for that minority of people who are the majority of clients. Couns Psychother Res.

[10] Yano K, Oishi K (2018). The relationships among daily exercise, sensory-processing sensitivity, and depressive tendency in Japanese university students. Pers Individ Dif.

[11] Booth C, Standage H, Fox E (2015). Sensory-processing sensitivity moderates the association between childhood experiences and adult life satisfaction. Pers Individ Dif.

[12] Listou Grimen H, Diseth Å (2016). Sensory processing sensitivity: Factors of the Highly Sensitive Person Scale and their relationships to personality and subjective health complaints. Percept Mot Skills.

[13] Meredith PJ, Bailey KJ, Strong J, Rappel G (2016). Adult attachment, sensory processing, and distress in healthy adults. Am J Occup Ther.

[14] Aron EN, Aron A (1997). Sensory-processing sensitivity and its relation to introversion and emotionality. J Pers Soc Psychol.

[15] Zigmond AS, Snaith RP (1983). The hospital anxiety and depression scale. Acta Psychiatr Scand.

[16] Pérez-Chacón M, Borda-Mas M, Chacón A, Avargues-Navarro ML (2023). Personality traits and coping strategies as psychological factors associated with health-related quality of life in highly sensitive persons. Int J Environ Res Public Health.

[17] Liss M, Mailloux J, Erchull MJ (2008). The relationships between sensory processing sensitivity, alexithymia, autism, depression, and anxiety. Pers Individ Dif.

[18] Grinapol S, Gelkopf M, Pagorek-Eshel S, Greene T (2022). The role of sensory processing sensitivity in the early traumatic stress reaction: Predicting post-traumatic stress symptoms following motor vehicle accidents. Pers Individ Dif.

[19] Pluess M, Boniwell I (2015). Sensory-processing sensitivity predicts treatment response to a school-based depression prevention program: Evidence of vantage sensitivity. Pers Individ Dif.

[20] Benham G (2006). The highly sensitive person: Stress and physical symptom reports. Personality and individual differences. Pers Individ Dif.

[21] Pérez-Chacón M, Chacón A, Borda-Mas M, Avargues-Navarro ML (2021). Sensory processing sensitivity and compassion satisfaction as risk/protective factors from burnout and compassion fatigue in healthcare and education professionals. Int J Environ Res Public Health.

